# Effects of early‐life exposure to THIP on brainstem neuronal excitability in the *Mecp2*‐null mouse model of Rett syndrome before and after drug withdrawal

**DOI:** 10.14814/phy2.13110

**Published:** 2017-01-20

**Authors:** Weiwei Zhong, Christopher M. Johnson, Ningren Cui, Max F. Oginsky, Yang Wu, Chun Jiang

**Affiliations:** ^1^ Department of BiologyGeorgia State UniversityAtlantaGeorgia

**Keywords:** Electrophysiology, gaboxadol, locus coeruleus, *Mecp2*, mesencephalic trigeminal, neuronal hyperexcitability

## Abstract

Rett syndrome (RTT) is mostly caused by mutations of the X‐linked *MECP2* gene. Although the causal neuronal mechanisms are still unclear, accumulating experimental evidence obtained from *Mecp2* ^−/Y^ mice suggests that imbalanced excitation/inhibition in central neurons plays a major role. Several approaches may help to rebalance the excitation/inhibition, including agonists of GABA_A_ receptors (GABA_A_R). Indeed, our previous studies have shown that early‐life exposure of *Mecp2*‐null mice to the extrasynaptic GABA_A_R agonist THIP alleviates several RTT‐like symptoms including breathing disorders, motor dysfunction, social behaviors, and lifespan. However, how the chronic THIP affects the *Mecp2* ^−/Y^ mice at the cellular level remains elusive. Here, we show that the THIP exposure in early lives markedly alleviated hyperexcitability of two types of brainstem neurons in *Mecp2* ^−/Y^ mice. In neurons of the locus coeruleus (LC), known to be involved in breathing regulation, the hyperexcitability showed clear age‐dependence, which was associated with age‐dependent deterioration of the RTT‐like breathing irregularities. Both the neuronal hyperexcitability and the breathing disorders were relieved with early THIP treatment. In neurons of the mesencephalic trigeminal nucleus (Me5), both the neuronal hyperexcitability and the changes in intrinsic membrane properties were alleviated with the THIP treatment in *Mecp2*‐null mice. The effects of THIP on both LC and Me5 neuronal excitability remained 1 week after withdrawal. Persistent alleviation of breathing abnormalities in *Mecp2* ^−/Y^ mice was also observed a week after THIP withdrawal. These results suggest that early‐life exposure to THIP, a potential therapeutic medicine, appears capable of controlling neuronal hyperexcitability in *Mecp2* ^−/Y^ mice, which occurs in the absence of THIP in the recording solution, lasts at least 1 week after withdrawal, and may contribute to the RTT‐like symptom mitigation.

## Introduction

Rett syndrome (RTT) is a neurodevelopmental disorder, caused mostly by mutations of the X‐linked *MECP2* gene, a transcriptional regulator. Patients with RTT, almost exclusively girls, develop various symptoms, such as stereotype behaviors, autism‐like social defects, motor dysfunctions, and life‐threatening breathing abnormalities (Zoghbi 2005). As a widely used RTT mouse model, *Mecp2* ^−/Y^ mice with *Mecp2* gene turned off in all cells recapitulate most of these RTT‐like symptoms and die in early ages (Guy et al. 2001). Besides uncovering RTT symptomatic and pathological changes, the mouse model is useful for finding potential therapeutic agents.

The studies in *Mecp2* ^−/Y^ mice suggest that imbalanced excitation/inhibition in the central nervous system (CNS) play a major role in the development of RTT. The altered excitation and inhibition have been found in multiple brain regions, including the brainstem (Taneja et al. 2009; Jin et al. 2013b; Oginsky et al. 2016) and the hippocampus (Calfa et al. 2015). In *Mecp2*‐null mice, excessive excitatory activity was seen in expiratory cranial and spinal nerves (Abdala et al. 2010). Neurons in the locus coeruleus (LC), the major norepinephrine (NE) source in the CNS, are overly excitable in *Mecp2*‐null mice, which is attributable to their defective intrinsic membrane properties and the reduced GABAergic inhibition, and may contribute to breathing abnormalities (Taneja et al. 2009; Zhang et al. 2010a; Jin et al. 2013a). Neurons in the mesencephalic trigeminal nucleus (Me5) nucleus, located adjacent to the LC, were found hyperexcitable as well (Johnson et al. 2016; Oginsky et al. 2016), which may contribute to the difficulties in chewing and eating in people with RTT (Isaacs et al. 2003; Motil et al. 2012).

The neuronal hyperexcitability involves the GABA system. Mice with *Mecp2* gene deletion selectively in the GABAergic neurons display RTT‐like phenotypes (Chao et al. 2010). Restoration of the gene in the *Mecp2*‐null GABAergic cells rescued these symptoms, including lifespan, social behaviors, and motor functions (Ure et al. 2016). Therefore, enhancing GABAergic inhibition may help to rebalance the excitation/inhibition in mouse models and perhaps human patients with RTT, leading to alleviation of the RTT‐like symptoms. Consistent with the idea, treatment with GABA reuptake blocker NO711 and synaptic GABA receptor agonist benzodiazepine relieves the RTT‐like breathing difficulties in *Mecp2*‐null mice (Abdala et al. 2010; Voituron and Hilaire 2011).

In *Mecp2*‐null LC neurons, both GABA_A_R and GABA_B_ receptors are deficient (Jin et al. 2013a). In contrast to the synaptic GABA_A_R, the expression level of extrasynaptic GABA_A_R is well maintained in *Mecp2*‐null LC neurons (Zhong et al. 2015). They may provide an alternative target to alter the excitation/inhibition balance. Indeed, we have recently shown that early treatment with THIP (also known as Gaboxadol), an extrasynaptic GABA_A_R agonist, alleviates the RTT‐like motor dysfunction, breathing abnormalities and the defects in social behaviors, expands the lifespan by enhancing the tonic GABAergic inhibition in *Mecp2*‐null mice (Zhong et al. 2016). However, several questions remain: How does the THIP treatment affect the *Mecp2*‐null mice at the cellular level? Will the systemic THIP treatment affect other brainstem neurons? Are the THIP effects lost totally after THIP clearance with withdrawal? Does a rebound excitation occur after THIP withdrawal? To address these questions, therefore, we performed the experiments.

## Materials and Methods

### Animals

Female heterozygous *Mecp2* ^+/−^ mice (Strain name: B6.129P2(C)‐*Mecp2*tm1.1Bird/J; Stock number 003890) from Jackson Lab were crossbred with wild‐type (WT) male C57BL/6 mice to produce the RTT model mice with the genotype *Mecp2* ^−/Y^. The PCR protocol from Jackson Lab was used to identify the genotype. All experimental procedures in mice were conducted in accordance with the National Institutes of Health (NIH) Guide for the Care and Use of Laboratory Animals and were approved by the Georgia State University Institutional Animal Care and Use Committee.

### THIP administration

The THIP exposure on *Mecp2* ^−/Y^ mice were described in previous study (Zhong et al. 2016). Briefly, THIP was given to the test animals orally in the drinking water. THIP was delivered to the mother in her drinking water at 200 mg/L, which passed to their pups via lactation, until weaning at P18, including both WT and *Mecp2* ^−/Y^ male pulps. After weaning, THIP was delivered to these male animals at 20 mg/L for another 5 weeks, and then these animals were continued to feed with regular drinking water until die.

### Brain slice preparation

Mice were decapitated after deep anesthesia with inhalation of saturated isoflurane. The brainstem was removed and immediately placed in the ice‐cold and sucrose‐rich artificial cerebrospinal fluid (aCSF) (in mM) containing 220 sucrose, 10 D‐glucose, 1.9 KCl, 0.5 CaCl_2_, 6 MgCl_2_, 33 NaHCO_3_ and 1.2 NaH_2_PO_4_. The solution was bubbled with 95% O_2_ balanced with 5% CO_2_ (pH 7. 40). The transverse pontine sections (150–250 *μ*m) containing the LC or Me5 area were obtained using a vibratome sectioning system. The slices were allowed to recover at 33°C for 60 min in normal aCSF (in mM) containing 10 D‐glucose, 124 NaCl, 3 KCl, 2 CaCl_2_, 2 MgCl_2_, 26 NaHCO_3_, and 1.3 NaH_2_PO_4_, and then maintained at room temperature before use. Upon recording, a brain slice was placed in the recording chamber and perfused with oxygenated aCSF at a rate of 2 mL/min and maintained at 34°C in a recording chamber by a dual automatic temperature control (Warner Instruments).

### Electrophysiology

Whole‐cell current clamp was performed on LC or Me5 neurons. Patch pipettes with resistance of 3–5 MΩ were pulled with Sutter pipette puller (Model P‐97, Novato, CA). Only were the neurons with membrane potential less than −40 mV (LC neurons) or −50 mV (Me5 neurons) and action potential (AP) larger than 65 mV accepted for further experiments. The pipette solution (in mM) contained 130 K gluconate, 10 KCl, 10 HEPES, 2 Mg‐ATP, 0.3 Na‐GTP and 0.4 EGTA (pH 7. 3). The bath solution was normal aCSF bubbled with 95% O_2_ and 5% CO_2_ (pH 7. 40). Recorded signals were amplified with an Axopatch 200B amplifier (Molecular Devices, Union City, CA), digitized at 10 kHz, filtered at 1 kHz, and collected with the Clampex 9 data acquisition software (Molecular Devices). Paired experiments were done in the patch clamp experiments and the data were obtained by three people double‐blindly.

In Me5 neurons, membrane potential of neurons was measured at resting status without any current injection. APs were evoked with depolarizing pulses. AP properties (threshold, amplitude, rise time, and D_50_) were analyzed from the first evoked APs.

### Plethysmograph recording

The plethysmograph system was composed of a ~40 mL plethysmograph chamber, where an unanesthetized mouse was housed. The chamber was connected to a reference chamber via a plastic tubing. With an individual animal, the plethysmograph chamber was flowed by atmospheric air at a rate of 60 mL/min for at least 20 min for adaptation followed by a 20 min recording. The breathing activity was recorded as the barometrical changes between the recording plethysmograph chamber and the reference chamber with a force‐electricity transducer (CB Sciences, Inc., Milford, MA). The signal was amplified and collected with Pclamp 9 software. The animals were monitored to ensure the awake status during tests. The data analysis was done blindly to the treatment. Apnea was considered only if the breathing cycle was twice longer than the previous one. Breathing frequency variation was calculated as the division of standard deviation (SD) of the frequency by their arithmetic mean. All the SD and arithmetic mean were measured from 150~200 successive breathing cycles, which were randomly sampled from three or four stretches with at least 50 breaths in each.

### Randomization

The animals used in the study were randomly separated into vehicle group and THIP group. The electrophysiology experiments were done by two to three people blindly to the mouse genotype and treatment.

### Data Analysis

The sample sizes in the experiments were examined with G‐Power Analysis to yield sufficient statistical power. Data are presented as means ± SE, Pearson's correlation, Student's t‐test, ANOVA, Tukey's post hoc, and *χ*2‐test were used to perform the statistical analysis. Difference was considered significant when *P* < 0.05.

## Results

### Age‐dependent hyperexcitability of LC and Me5 neurons in *Mecp2* ^−/Y^ mice

All experiments were done in male *Mecp2* ^−/Y^ mice because the males offer a completely *Mecp2*‐null condition that is not always available in *Mecp2* ^+/−^ females owing to X‐chromosome inactivation.

**Figure 1:**
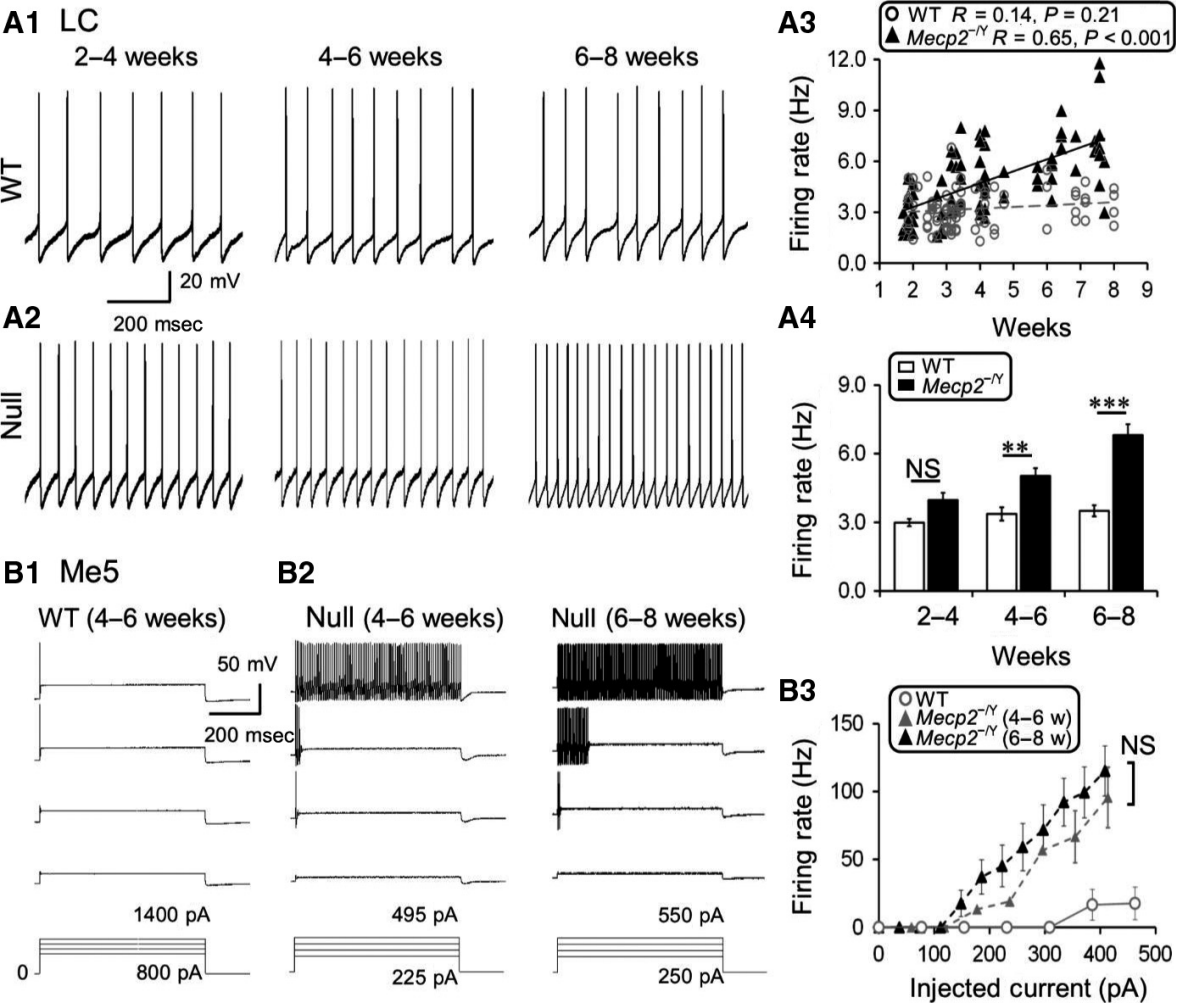
Age‐dependent increase in excitability of locus coeruleus (LC) and Me5 neurons in *Mecp2* ^−/Y^ mice. (A_1_‐A_2_) Neuronal activity was studied in whole‐cell current clamp, LC neurons in *Mecp2* ^−/Y^ mouse showed increased firing frequency in comparison to its wild‐type (WT) counterpart. Such hyperexcitability deteriorated with growth. (A_3_‐A_4_) Statistically, the increased LC neuronal excitability in *Mecp2*‐null mice was significantly different from the WT, and showed age dependence (A_3_: Pearson correlation. A_4_: Significant differences were found in the main factors of genotype (df = 1, *F* = 66.14, *P *<* *0.001) and age (df = 1, *F* = 16.53, *P *<* *0.001). Significant interaction was found between the two factors as well. ### *P* < 0.01; Two‐way ANOVA and **P <* *0.01, ***P <* *0.001; Tukey's post hoc). (B_1_‐B_2_) With injections of a series of depolarizing currents, most of the Me5 neurons in WT neurons fired single action potential, while the *Mecp2*‐null Me5 ones fired multiple action potentials. The excitability of Me5 neurons in *Mecp2*‐null did not show the age dependence (NS, not significantly different; Student's t‐test).

Previous studies indicate that LC neurons are overly excitable in *Mecp2*‐null mice compared to the WT (Taneja et al. 2009; Zhang et al. 2010a). To show how such hyperexcitability progresses with age, we studied LC neuronal excitability in three age groups of WT and *Mecp2*‐null mice. Our results showed that such neuronal hyperexcitability was age‐dependent (Fig. 1A_1‐2_). When the spontaneous firing rate of LC neurons was plotted against ages, a linear age‐dependent increase in the firing rate is seen, in which the regression is significant in *Mecp2*‐null but not WT mice (Fig. 1A_3_; WT: R = 0.14, *n* = 82, *P *=* *0.21; *Mecp2*‐null: *R* = 0.65, *n* = 79, *p* < 0.001; WT versus *Mecp2*‐null: *P *=* *0.001). A significant increase in spontaneous firing activity of LC neurons started at 2–4 weeks, and became more obvious at age 4–6 weeks. The neuronal firing rate doubled that of the WT at 6–8 weeks (Fig. 1A_4_; 2–4 weeks: *n* = 45 and *n* = 38; 4–6 weeks: *n* = 17 and *n* = 20; 6–8 weeks: *n* = 12 and *n* = 21; WT and *Mecp2*‐null, respectively). Both are consistent with the onset time of RTT‐like symptoms and the age‐dependent symptom deterioration of *Mecp2*‐null mice. In contrast, the age‐dependent increase in LC neuronal excitability was not observed in WT mice (Fig. 1A_4_).

Me5 neurons were silent at basal condition without current injection in both WT and *Mecp2*‐null mice. In response to depolarizing current injection, the Me5 neurons in *Mecp2*‐null mice tended to fire multiple action potentials (APs) in comparison to one or two APs in their WT counterparts (Fig. 1B_1_‐B_2_). With comparable levels of current injection, Me5 neurons showed significantly higher firing rate in *Mecp2*‐null mice than in the WT, indicating that they also are hyperexcitable. Unlike LC neurons, the Me5 neuronal hyperexcitability did not show significant age dependence (Fig. 1A_4_; WT: *n* = 14; *Mecp2*‐null 4–6 weeks: *n* = 17; *Mecp2*‐null 6–8 weeks: *n* = 14; Fig. 1B_3_).

### Relationship of LC neuronal excitability with breathing abnormalities

**Figure 2:**
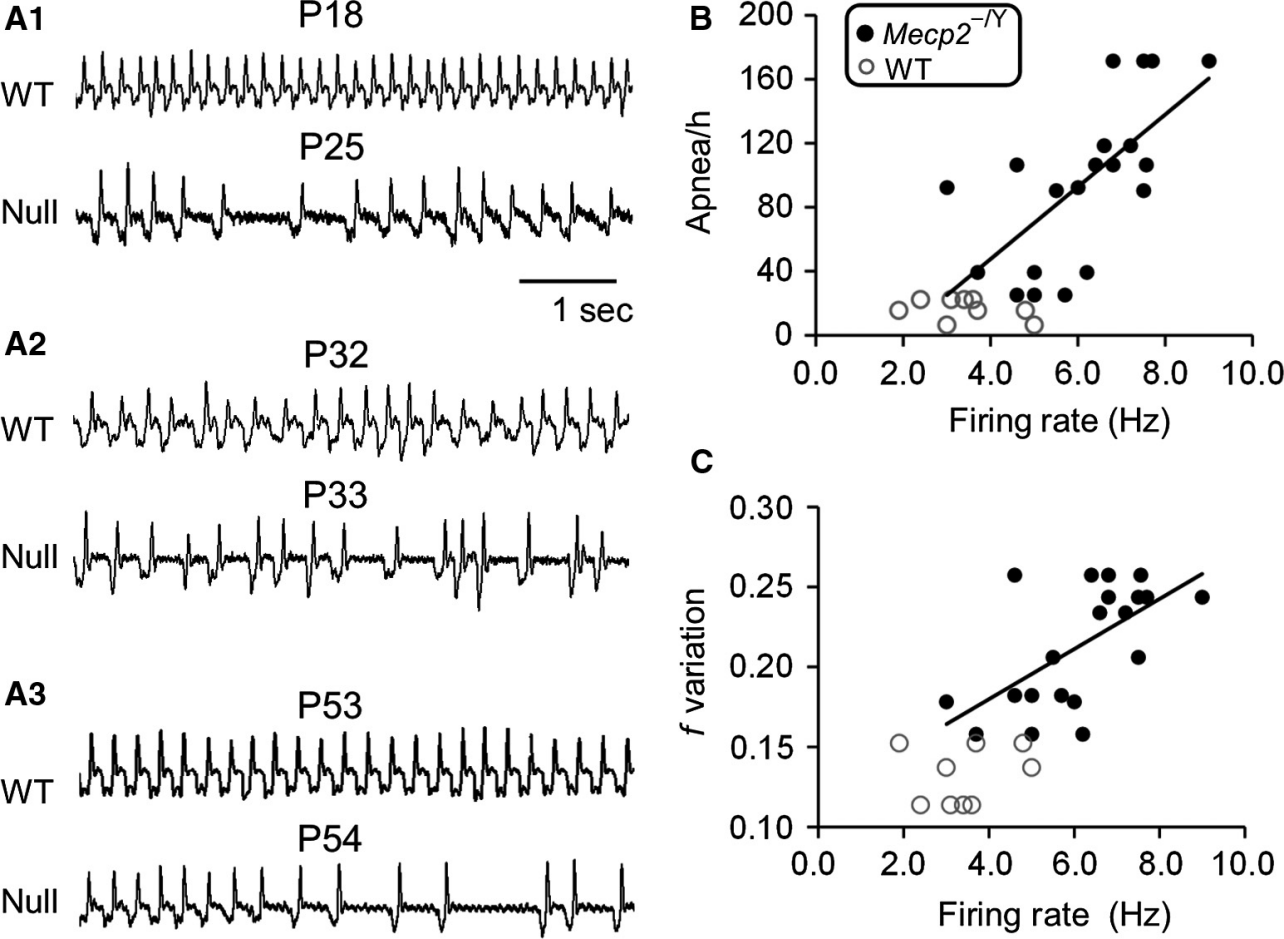
Relationship of locus coeruleus (LC) neuronal excitability with breathing abnormalities (A_1_‐A_3_) Typical records of breathing activity from both wild‐type(WT) and *Mecp2*‐null mice at different ages and the breathing abnormalities deterioration with the age. (B‐C) Breathing activity was measured immediately before *Mecp2*‐null mice were used for brain slice studies. In the *Mecp2*‐null mice older than 6 weeks, LC neuronal firing rate increased proportionally with the severities of apnea rate and breathing frequency variation (Pearson correlation). Such relationship was not found in the WT mice.

It is possible that inhibition of LC neuronal hyperexcitability by THIP may affect breathing abnormalities, as LC neurons play a role in breathing regulation, and as the age‐dependent deterioration was found the *Mecp2*‐null mice in our previous studies (Zhong et al. 2016), Therefore, we performed electrophysiological recording from in 6–8‐week‐old mice whose breathing activity was measured on the same day immediately before euthanasia (Fig. 2A). When LC neuronal firing rate was plotted against apnea rate (Fig. 2B, *n* = 20) or breathing frequency variation (Fig. 2C, *n* = 20), we found that the LC neuronal firing rate increases proportionally with the severity of these breathing abnormalities. Both can be described with a linear regression (*Mecp2*‐null: Fig. 2B: *R* = 0.66, *P *<* *0.01; 2C: *R* = 0.62, *P *<* *0.01). Such proportional changes in firing rate with breathing abnormalities were not seen in WT neurons (Fig. 2B,C).

### Mitigation of LC and Me5 neuronal hyperexcitability with THIP exposure

**Figure 3:**
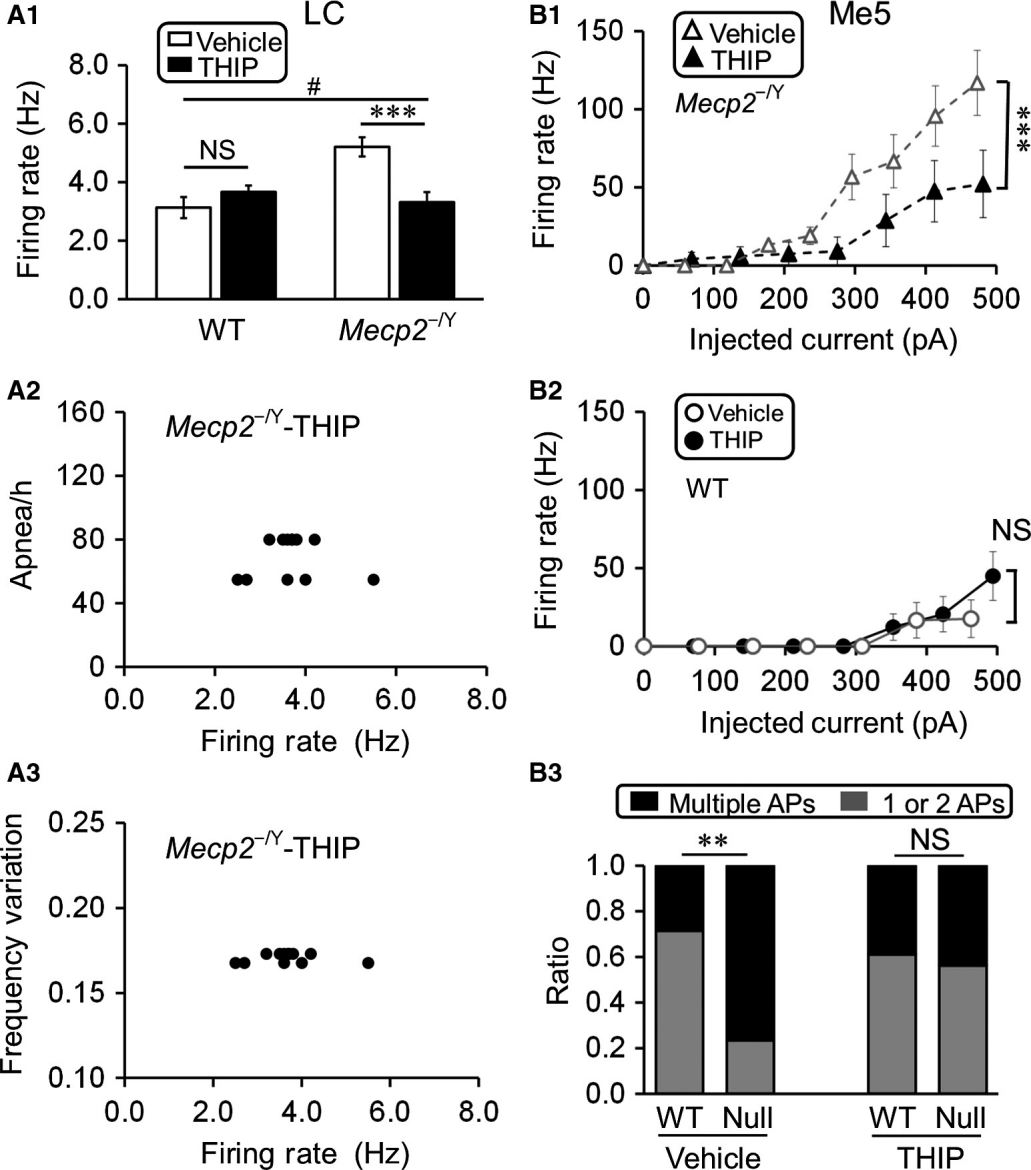
THIP exposure alleviated the locus coeruleus (LC) and Me5 neuronal hyperexcitability. (A_1_) Administration of THIP significantly reduced the hyperexcitability of LC neurons in *Mecp2*‐null mice. Significant differences were found in the main factors of genotype (df = 1, *F* = 8.14, *P *=* *0.006) and treatment (df = 1, *F* = 4.49, *P *=* *0.040). Significant interaction was found between the two factors as well (#*P *<* *0.05; Two‐way ANOVA and ***P <* *0.001; Tukey's post hoc). (A_2_‐A_3_) No significant correlation was found between the LC spontaneous firing rate and the apnea rate (A_2_)/breathing frequency variation (A_3_) (Pearson correlation). (B) THIP significantly reduced the firing rate of Me5 neurons in *Mecp2*‐null mice with comparative amount of current injections (B_1_). Such a relief of Me5 neuronal excitability was not found in the wild‐type (WT) mice (B_2_). In comparison to the WT, a significantly larger number of Me5 neurons in *Mecp2*‐null mice fired multiple action potentials, which was also suppressed by THIP treatment (B_3_) (*** *P *<* *0.001, ** *P *<* *0.01; Student's t‐test and *χ*2‐test).

To test how the extrasynaptic GABA_A_R agonist THIP affects these brainstem neurons, WT and *Mecp2*‐null mice were exposed to THIP in their drinking water as described in the Methods. With continuing THIP treatment for 5~6 weeks starting from birth, brain slices were obtained from the mice without drug withdrawal, in which neuronal activity was studied. In *Mecp2*‐null mice the spontaneous firing rate of LC neurons was stabilized to the level of WT neurons, which was significantly lower than the vehicle control (Fig. 3A_1_; WT: 3.1 ± 1.3 Hz and 3.7 ± 0.8 Hz, *n* = 13 and *n* = 12, *P* = 0.602; *Mecp2*‐null: 5.2 ± 1.2 Hz and 3.3 ± 1.2 Hz, *n* = 13 and *n* = 12; vehicle and THIP, respectively). Note that no THIP was added to the recording solutions.

Our previous studies suggest THIP treatment significantly reduced the apnea rate and breathing frequency variation in the *Mecp2*‐null (Zhong et al. 2016). To show the relationship these breathing abnormalities with the reduced LC neuronal excitability, LC neuronal firing rate was plotted against apnea rate (Fig. 3A_2_, *n* = 12) or breathing frequency variation in the *Mecp2*‐null mice treated with THIP (Fig. 3A_3_, *n* = 12) similarly as in Fig. 2B,C. In these plots, no significant correlation was seen (*R* = 0.01, *P *>* *0.05 in 3A_2_, *R* = 0.01, *P *>* *0.05 in A_3_).

A similar excitability relief was found in Me5 neurons of *Mecp2*‐null mice (Fig.3B). The evoked firing activity with depolarizing current injection was significantly lower in Me5 cells from THIP‐treated *Mecp2*‐null mice than the vehicle‐treated (Fig. 3B_1_; vehicle: *n* = 14; THIP: *n* = 18). No significant difference in Me5 neuronal firing rates was found between the THIP‐ and vehicle‐treated WT (Fig. 3B_2_). With the current injection, some Me5 cells fired repetitive APs. The ratio of cells with multiple APs versus those with one or two APs was significantly higher in *Mecp2*‐null mice than in the WT. Such a difference was abolished with the THIP treatment (Fig. 3B_3_; vehicle: *n* = 14 and *n* = 17; THIP: *n* = 18 and *n* = 16; WT and *Mecp2*‐null, respectively). Together, these results suggest that early‐life THIP exposure significantly suppressed the hyperexcitability of both LC and Me5 neurons in *Mecp2*‐null mice.

### THIP effects on intrinsic membrane properties of null Me5 neurons

The early‐life exposure to THIP may affect these brainstem neurons by changing their intrinsic membrane properties. Thus, we performed detailed studies of subthreshold and suprathreshold properties. Since we had done similar studies in LC neurons before (Zhang et al. 2010a; Johnson et al. 2016), we were focused on Me5 neurons in this study.

In the Me5 neurons, AP amplitude was measured from its threshold level to the peak. The rise time of AP was measured as the period from the AP threshold to the peak. AP width (D_50_) measured as the width at half AP amplitude. To measure the resistance, Sag and postinhibitory rebound (PIR), neurons were injected with a series of hyperpolarizing currents. After the termination of each command, the cell responded with a postinhibitory depolarization or AP (when the rebound reached AP threshold). The three parameters were calculated based on the trace immediately before the AP was initiated. The input resistance was measured as the ratio of steady‐state voltage at the command current. The sag was calculated as the difference between the peak hyperpolarization during the current injection and the steady‐state potential. The PIR was defined as the difference between the peak depolarization of the rebound and the resting membrane potential.

**Figure 4:**
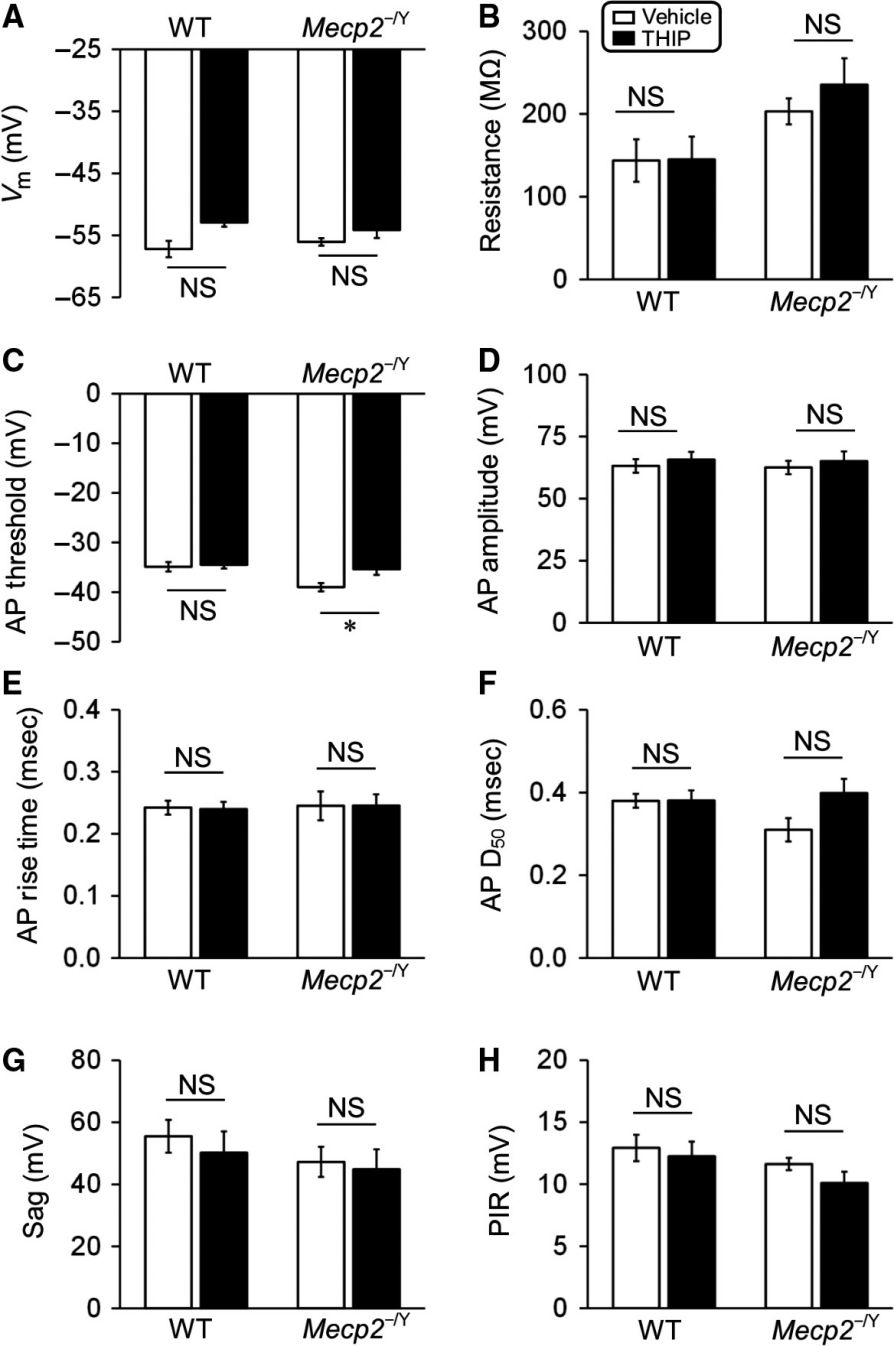
THIP effects on intrinsic membrane properties of *Mecp2*‐null Me5 neurons. (A–B) The membrane potential and input resistance were not altered with THIP treatment in either *Mecp2*‐null Me5 cells or wild‐type (WT) ones. (C–F) THIP significantly shifted the firing threshold of Me5 neurons to more depolarizing potentials in *Mecp2*‐null mice, but not in WT (C). THIP did not change the other parameters of action potential (AP) morphology, including amplitude, rise time and half width (D_50_, measured at 50% amplitude) in either WT or *Mecp2*‐null neurons (D‐F). (G–H) In Me5 cells, Sag and postinhibitory rebound (PIR) was not significantly changed with THIP treatment as well (* *P *<* *0.05; Student's t‐test).

Similar to LC neurons, the chronic THIP did not show significant effects on the membrane potential, input resistance, AP properties (amplitude, rise time, and D_50_), Sag and PIR of Me5 neurons (Fig. 4), whereas the firing threshold of Me5 neurons in *Mecp2*‐null mice was shifted to more depolarizing potentials with THIP treatment, which was significant in comparison to the vehicle control (Fig. 4C). Therefore, in *Mecp2*‐null mice, early treatment of THIP seems to raise firing threshold without affecting other intrinsic membrane properties of Me5 neurons.

### Persistent inhibition of neuronal excitability 1‐week after THIP withdrawal

The suppression of brainstem neuronal excitability may be affected by THIP withdrawal, as rebound excitation usually merges with withdrawal of certain neuronal suppressants. Therefore, we studied neuronal activity after THIP withdrawal. To avoid the likelihood that residue THIP may exist in the body, we chose to do the experiments 7 days after withdrawal.

**Figure 5:**
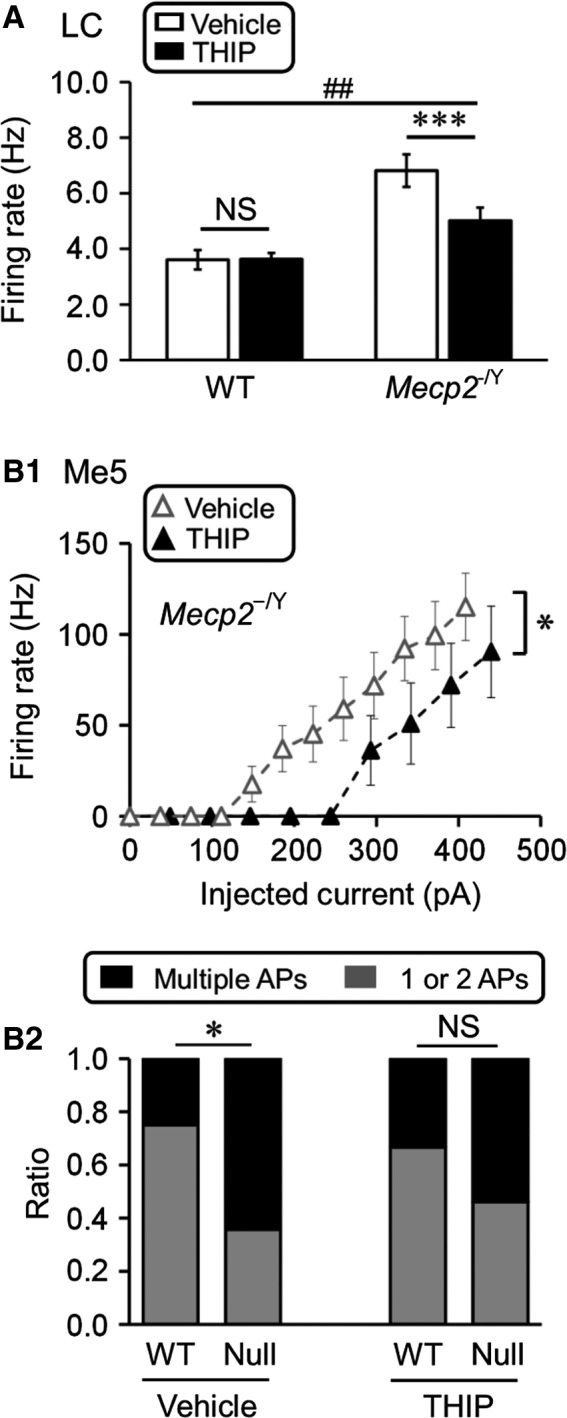
The THIP effects on neuronal excitability 1 week after withdrawal. (A) The hyperexcitability of LC neurons was significantly lower in *Mecp2*‐null mice with THIP treatment than in those without. Significant differences were found in the main factors of genotype (df = 1, *F* = 34.27, *P *<* *0.001) and treatment (df = 1, *F* = 7.55, *P *=* *0.008). No significant interaction was found between the two factors (##*P *<* *0.01, Two‐way ANOVA and ***P <* *0.001, Tukey's post hoc). (B) In *Mecp2*‐null Me5 neurons, the suppression of neuronal excitability by THIP was remained (B_1_). The number of cells with repetitive firing activity was significantly reduced with THIP treatment in comparison to those without (B_2_) (*P <* *0.05; Student's t‐test and *χ*2‐test).

The firing rate of LC neurons in THIP‐treated mice remained significantly lower than that of the vehicle control (Fig. 5A; WT: 3.6 ± 0.8 Hz and 3.6 ± 1.2 Hz, *n* = 14, and *n* = 12; *Mecp2*‐null: 6.8 ± 2.2 Hz and 5.0 ± 1.6 Hz, *n* = 21 and *n* = 12; vehicle and THIP, respectively). No rebound excitation was found in Me5 neurons either. Instead, the evoked firing rate of Me5 neurons from THIP‐treated *Mecp2*‐null mice was significantly lower than that of vehicle control a week after THIP withdrawal (Fig. 5B_1_). Furthermore, the ratio of Me5 cells with versus without multiple APs remained about the same between *Mecp2*‐null and WT mice, in comparison to the significant difference in the ratio between vehicle controls (Fig. 5B_2_). These results suggest that THIP withdrawal does not seem to cause rebound excitation. Instead, the THIP effects seem persistent, as both LC and Me5 neurons of *Mecp2*‐null mice retained their excitability similar to their WT counterparts 1 week after THIP withdrawal (Fig. 5B_1_–B_2_; vehicle: *n* = 14 and *n* = 17; THIP: *n* = 9 and *n* = 13; WT and *Mecp2*‐null, respectively).

### Breathing abnormalities remained suppressed one‐week after THIP withdrawal

Our previous study has shown that the same THIP treatment significantly suppressed the breathing abnormalities (Zhong et al. 2016). We thus studied how breathing was affected by THIP withdrawal.

**Figure 6:**
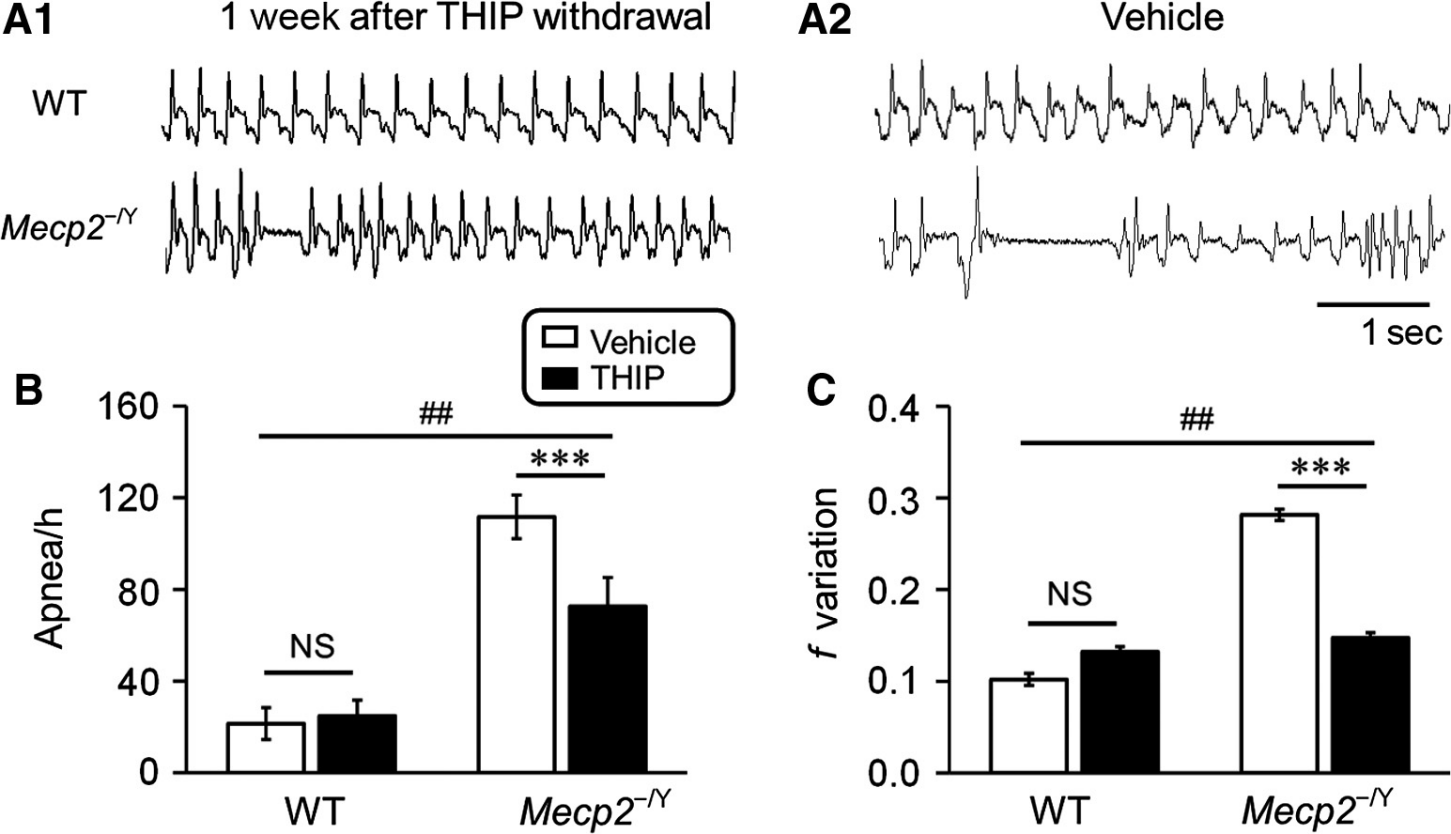
Breathing abnormalities remained suppressed one‐week after THIP withdrawal. (A_1_–A_2_) Typical records of breathing activity from both wild‐type (WT) and *Mecp2*‐null mice at P61. (B–C) The apnea rate (B) and breathing frequency variation (C) in *Mecp2*‐null mice were significantly reduced in comparison to the vehicle control. Significant differences were found in the main factors of genotype (B: df = 1, *F* = 57.89, *P *<* *0.001; C: df = 1, *F* = 34.44, *P *<* *0.001) and treatment (B: df = 1, *F* = 8.11, *P *=* *0.007; C: df = 1, *F* = 10.33, *P *=* *0.003). Significant interactions were found between the two factors in apnea, but not in breathing frequency variation (##*P *<* *0.01, Two‐way ANOVA and ***P <* *0.001, Tukey's post hoc).

One week after THIP withdrawal, both apnea events and breathing frequency variation in *Mecp2*‐null mice remained lower than the vehicle control (Fig. 6A_1_–A_2_). Statistical analysis showed that in *Mecp2*‐null mice, the apnea rate and breathing frequency variation were significantly lower in mice that had been treated with THIP than those treated with vehicle (Fig. 6B–C; WT: *n* = 17 and *n* = 3; *Mecp2*‐null: *n* = 19 and *n* = 8; sham and THIP, respectively). These results, consistent with the prolonged neuronal excitability depression, indicate that the effects of THIP treatment seem to persist at least for 1 week after THIP withdrawal without apparent rebound excitation.

## Discussion

This is the first study of the cellular outcome of early‐life exposure to THIP. We have found that the THIP exposure markedly alleviates hyperexcitability of two types of brainstem neurons in *Mecp2* ^−/Y^ mice. In LC neurons known to be involved in breathing regulation, the hyperexcitability shows clear age‐dependence associated with age‐dependent deterioration of the RTT‐like breathing irregularities, both of which are relieved with early THIP treatment. In Me5 neurons of *Mecp2*‐null mice, the hyperexcitability as well as the changes in intrinsic membrane properties are both improved with the THIP treatment. One week after THIP withdrawal, excitability of both LC and Me5 neurons remained depressed. Consistent with the proportional relationship between LC firing rate and breathing irregularities and the persistent effects of THIP on cellular excitability, RTT‐like breathing abnormalities of *Mecp2*‐null mice remain low in the time period after THIP withdrawal.

*Mecp2*‐null mice start to display a range of RTT‐like symptoms, including mobility problems and breathing difficulties around 3 weeks after birth, and most of the animals die within 2 months of age (Guy et al. 2001). The symptom development is consistent with the onset and deterioration of the neuronal hyperexcitation, especially LC neurons as shown in this study. Indeed, our results have shown that LC neuronal excitability increases proportionally with the severity of breathing abnormalities. Thus, the defects in LC neuronal excitability may play a role in the development of the RTT‐like symptoms in the mouse model. Consistent with this idea, the chronic THIP stabilizes LC neuronal excitability and breathing abnormalities to a similar degree (Fig. 3A). Also consistent with the idea are our recent studies showing that by enhancing the tonic GABAergic inhibition, early‐life exposure of *Mecp2*‐null mice to THIP alleviates various RTT‐like symptoms and extends lifespan (Zhong et al. 2016).

Although THIP may affect other brain regions by augmenting local extrasynaptic GABA_A_R, the stabilization of LC neuronal excitability may benefit a range of target regions. The LC is the major NE source in the CNS. The homeostasis of the LC neuronal excitability versus the NE synthesis, ensures the persistent production and release of NE. LC neuronal hyperexcitability may interrupt this balance and lead to impairment of the NE system, including the reduced expression of rate‐limiting enzyme, tyrosine hydroxylase (TH) and dopamine beta hydroxylase (DBH), and reduced NE concentration in the CNS (Zoghbi et al. 1985; Taneja et al. 2009; Roux et al. 2010; Zhang et al. 2010a,b; Zhang et al. 2016). A relief of LC hyperexcitability may reinstall the homeostatic state in the cells and improve NE output, which may benefit the LC‐NE projected target regions, including the brainstem, the spinal cord and the prefrontal cortex, brains areas critical for breathing, motor function, and social behaviors (Zhang et al. 2014; Schwarz et al. 2015).

The hyperexcitation is not limited to LC neurons. Our results have shown that Me5 neurons are also hyperexcitable (Oginsky et al. 2016). The Me5 neurons are the only group of propriosensory neurons with soma located in the CNS, which provide servo feedback control to the jaw muscles. The increased excitability in these neurons may impair these cranial muscles, consistent with the defects of chewing, drinking, speaking, and teeth grinding in people with RTT (Isaacs et al. 2003; Motil et al. 2012; Johnson et al. 2016; Oginsky et al. 2016). Suppression of overly excited Me5 neurons may lead to a correction in the proprioception of muscles, leading to the improvement of motor function in RTT. The Me5 neuronal hyperexcitability may be attributable to the impaired intrinsic membrane properties in *Mecp2*‐null mice.

Our results show that early‐life THIP exposure suppresses the neuronal hyperexcitability without affecting most of the intrinsic membrane properties, which suggests that THIP seems to restore the normal function of *Mecp2*‐null neurons via presynaptic inhibition. However, the firing threshold is shifted to more depolarizing potentials in both LC and Me5 neurons, indicating THIP may also alter postsynaptic mechanisms such as Na^+^ channel expressions that have been shown abnormal in *Mecp2*‐null mice (Zhang et al. 2010a; Oginsky et al. 2016).

In contrast to the neuronal hyperexcitability, hypoexcitability is found in certain forebrain neurons, which may contribute to the impaired sensorimotor gating function in RTT (Kron et al. 2012). Interestingly, inhibition of the NMDA receptors with ketamine can reverse the neuronal hypoexcitability and improve associated behaviors in *Mecp2*‐null mice, likely by disinhibiting cortical pyramidal cells (Kron et al. 2012; Patrizi et al. 2016). Apparently, excessive excitation may also be a problem in the cortical neuronal networks, which responds to NMDA receptor antagonism. Besides ketamine, enhancing the GABAergic inhibition might benefit hypoexcitability of certain forebrain neurons in *Mecp2*‐null mice as well.

THIP was previously tested as a potential clinical medicine for insomnia. With a short half‐life time around half an hour, the THIP concentration in the plasma diminished within 3 h (Cremers and Ebert 2007). However, continuous oral treatment may allow the THIP to remain at a certain level for a long period in the plasma and CNS, leading to the persistent effects on neurons and the associated phenotypes.

Due to the fast decay of the THIP, the withdrawal for 1 week may allow a total clearance of THIP from the plasma and the CNS. This time period seems adequate for evaluation of the persistence of its effects. Our results indicate that the THIP effects on relieving neuronal hyperexcitability remain 1 week after THIP withdrawal, which suggest chronic treatment of THIP may alter the expression of certain proteins involving the rebalance of excitation versus inhibition in the CNS. The results are consistent with the expanded lifespan in the THIP‐treated *Mecp2*‐null mice (Zhong et al. 2016). Although neuronal activity and breathing abnormalities remained alleviated with the THIP withdrawal in *Mecp2*‐null mice, their absolute levels were higher than the WT, indicating a gradual decline of the THIP effects.

Our results do not support the presence of rebound excitation 1 week after THIP withdrawal. However, the results cannot rule out the possibility of rebound excitation during the period of 1–6 days after THIP withdrawal. Because of the availability of these *Mecp2*‐null mice, we could not perform these follow‐up studies. Thus, further studies are needed to reveal the time course 1–6 days after THIP withdrawal.

In conclusion, cellular hyperexcitability has been found in multiple neurons of *Mecp2*‐null mice, associated with RTT‐like symptoms. Early‐life treatment with THIP reduces significantly the hyperexcitability of both LC and Me5 neurons in *Mecp2*‐null mice without affecting most of the intrinsic membrane properties. The THIP effect persists for at least 1 week after THIP withdrawal. The results suggest that THIP, a potential therapeutic medicine, seems capable of stabilizing neuronal excitability in *Mecp2*‐null mice, which may contribute to the RTT‐like symptom mitigation.

## Conflict of Interest

None declared.
